# Evaluation of *Candida* peritonitis with underlying peritoneal fibrosis and efficacy of micafungin in murine models of intra-abdominal candidiasis

**DOI:** 10.1038/s41598-019-45776-x

**Published:** 2019-06-27

**Authors:** Nobuyuki Ashizawa, Taiga Miyazaki, Shinichi Abe, Takahiro Takazono, Tomomi Saijo, Yoko Obata, Shintaro Shimamura, Kazuko Yamamoto, Yoshifumi Imamura, Takehiko Koji, Tomoya Nishino, Koichi Izumikawa, Katsunori Yanagihara, Shigeru Kohno, Hiroshi Mukae

**Affiliations:** 1 Department of Respiratory Medicine, Nagasaki University Graduate School of Biomedical Sciences, 1-7-1 Sakamoto, Nagasaki, Japan; 2 Department of Respiratory Medicine, Nagasaki University Hospital, 1-7-1 Sakamoto, Nagasaki, Japan; 3 Department of Infectious Diseases, Nagasaki University Graduate School of Biomedical Sciences, 1-7-1 Sakamoto, Nagasaki, Japan; 4 Department of Nephrology Medicine, Nagasaki University Graduate School of Biomedical Sciences, 1-7-1 Sakamoto, Nagasaki, Japan; 5 Medical Education Development Center, Nagasaki University Hospital, 1-7-1 Sakamoto, Nagasaki, Japan; 6 Department of Histology and Cell Biology, Nagasaki University Graduate School of Biomedical Sciences, 1-12-4 Sakamoto, Nagasaki, Japan; 7 Department of Laboratory Medicine, Nagasaki University Hospital, 1-7-1 Sakamoto, Nagasaki, Japan

**Keywords:** Infection, Fungal pathogenesis

## Abstract

*Candida* peritonitis is a crucial disease, however the optimal antifungal therapy regimen has not been clearly defined. Peritoneal fibrosis (PF) can be caused by abdominal surgery, intra-abdominal infection, and malignant diseases, and is also widely recognized as a crucial complication of long-term peritoneal dialysis. However, the influence of PF on *Candida* peritonitis prognosis remains unknown. Here, we evaluated the severity of *Candida* peritonitis within the context of PF and the efficacy of micafungin using mice. A PF mouse model was generated by intraperitoneally administering chlorhexidine gluconate. *Candida* peritonitis, induced by intraperitoneal inoculation of *Candida albicans*, was treated with a 7-day consecutive subcutaneous administration of micafungin. *Candida* infection caused a higher mortality rate in the PF mice compared with the control mice on day 7. Proliferative *Candida* invasion into the peritoneum and intra-abdominal organs was confirmed pathologically only in the PF mice. However, all mice in both groups treated with micafungin survived until day 20. Micafungin treatment tends to suppress inflammatory cytokines in the plasma 12 h after infection in both groups. Our results suggest that PF enhances early mortality in *Candida* peritonitis. Prompt initiation and sufficient doses of micafungin had good efficacy for *Candida* peritonitis, irrespective of the underlying PF.

## Introduction

Peritoneal dialysis (PD) is an effective treatment for end-stage renal disease^1–3^; however, several complications occur in patients undergoing PD. Peritoneal fibrosis (PF) is a pathological change occurring in the peritoneal membrane induced by long-term PD, which impairs the efficiency of dialysis and results in withdrawal from PD^4^. Infective peritonitis is also a common problem occurring in PD patients, which can be crucial. Bacteria, such as staphylococcal species, are common causative organisms of peritonitis^5,6^, but fungi, especially *Candida* species, are also important pathogens, which lead to high mortality rate of up to ≥25%^7–11^. *Candida albicans* is the most common species detected in >50% of cases^10,11^. Nevertheless, the optimal antifungal therapy regimen has not been clearly defined.

For fungal peritonitis, the International Society for Peritoneal Dialysis (ISPD) guideline updated in 2016, advocates for immediate catheter removal and antifungal therapy; however, the appropriate antifungal agent, for the initial therapy, has not been clearly defined^12^. Intra-abdominal candidiasis, such as peritonitis or abscesses, is considered much more common than recognized^13^. The causes of intra-abdominal candidiasis are not only from PD but also from intra-abdominal surgery, anastomotic leakage, and pancreatitis^14^. The Infectious Disease Society of America (IDSA) guideline on the management of candidiasis, updated in 2016, recommends echinocandin as an initial therapy for intra-abdominal candidiasis irrespective of the presence or absence of PF^14^. Micafungin is one of the antifungal agents belonging to the echinocandins class; and has been shown to have good antifungal activity even against non-*albicans Candida* species^15,16^. Non-*albicans Candida* species have recently increased as causative organisms in candidiasis patients^17–19^. However, *C*. *albicans* remains the predominant species causing *Candida* peritonitis. Furthermore, echinocandins are easy to use, with minimal adverse effects^20–22^. For these reasons, the clinical use of micafungin has recently increased.

PF and *Candida* peritonitis, two complications in PD patients, have been evaluated thus far; and severe or prolonged infective peritonitis is known to induce PF^23,24^. Yet, the influence of PF on peritoneal infection has not been adequately investigated. Therefore, evaluation of the efficacy of antifungal therapy for *Candida* peritonitis developed in the context of PF is essential among PD patients who suffer from *Candida* peritonitis. Furthermore, it could be useful for treating patients with *Candida* peritonitis in the context of abdominal surgery, intra-abdominal infection, and malignant diseases who may develop PF^25–27^. In this study, we evaluated whether PF has any influence on the prognosis of *Candida* peritonitis and on the efficacy of micafungin treatment, using PF mouse models.

## Results

### Evaluation of PF

**Figure 1:**
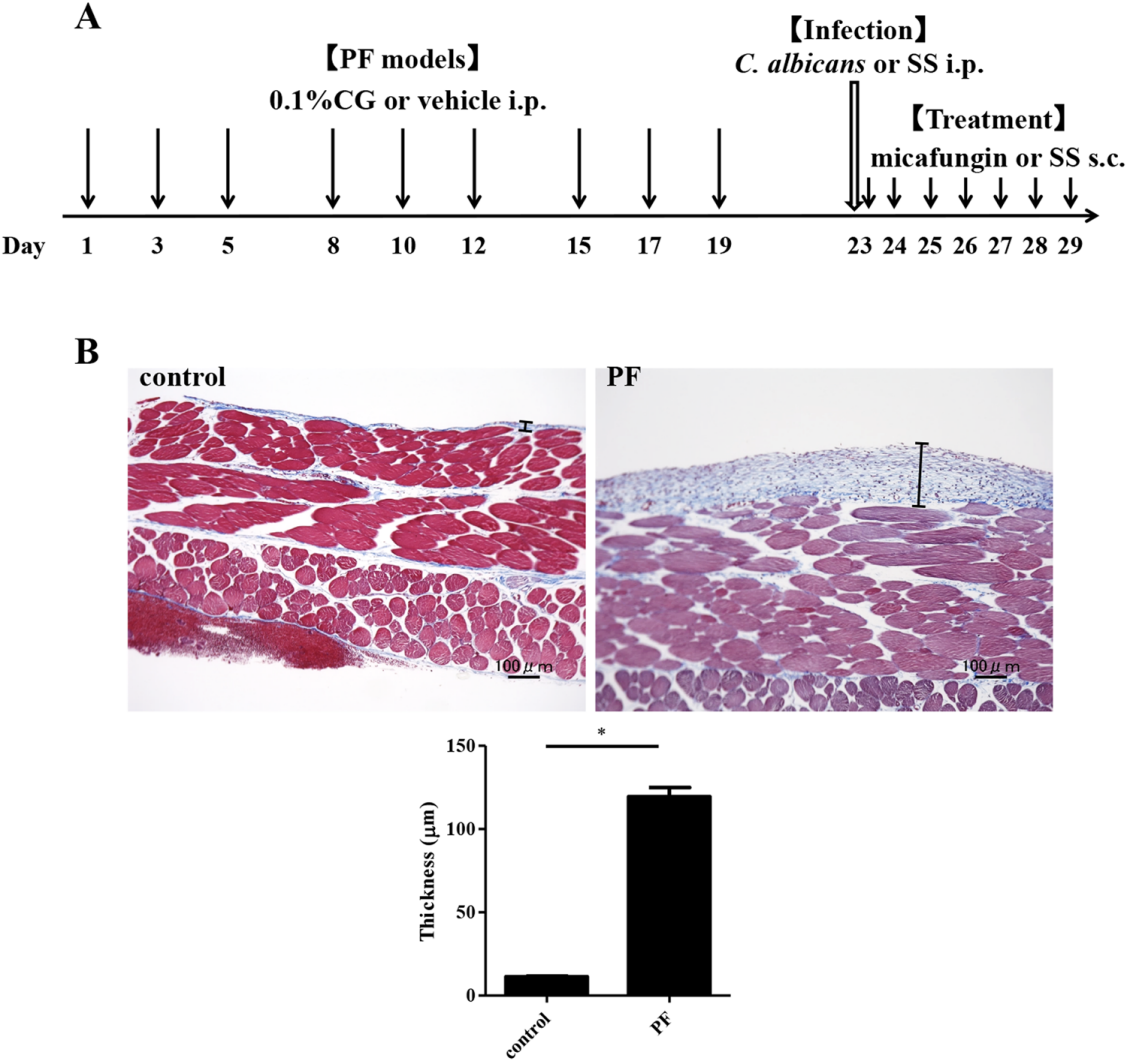
Schematic study design of the present study and histopathological evaluation of PF. (**A**) Mice received 9 intraperitoneal injections of 0.1% CG or a vehicle (15% ethanol) at a dose of 0.2 mL/mouse in 3 weeks, followed by one injection with 1 mL of the *C*. *albicans* cell suspension (5.0 × 10^7^ CFU/mouse). Micafungin dissolved in sterile saline was subcutaneously administered at a dose of 5 mg/kg daily for 7 days, beginning 2 h after the infection. (**B**) Masson’s trichrome staining of peritoneal tissues at 100-fold magnification. Bars indicate the thickness of the submesothelial compact zone. In the control group, the monolayer of mesothelial cells covered the entire surface of the peritoneum. In the PF group, the submesothelial compact zone was markedly thickened. Bar graph shows the thickness of the submesothelial compact zones in the control and PF groups. The PF group showed significant thickening compared to the control group. An asterisk indicates p < 0.0001 (unpaired t test). Both control and PF groups in this assay were not inoculated with *Candida*. These results were confirmed on two separate occasions and representative data are shown. PF, peritoneal fibrosis; CG, chlorhexidine gluconate; SS, sterile saline; i.p., intraperitoneal injection; and s.c., subcutaneous injection.

PF was experimentally induced in mice by repeated intraperitoneal administration of chlorhexidine gluconate (CG) (Fig. 1A). In the control group, the mice peritoneal tissue consisted of a peritoneal mesothelial monolayer, with sparse connective tissues below the layer. Compared to the control group, the peritoneal tissues of the mice in the PF group showed significant thickening of the submesothelial compact zone (p < 0.0001, unpaired t test), and the presence of numerous inflammatory cells and fibroblasts as previously reported^4^ (Fig. 1B).

### Evaluation of survival

**Figure 2:**
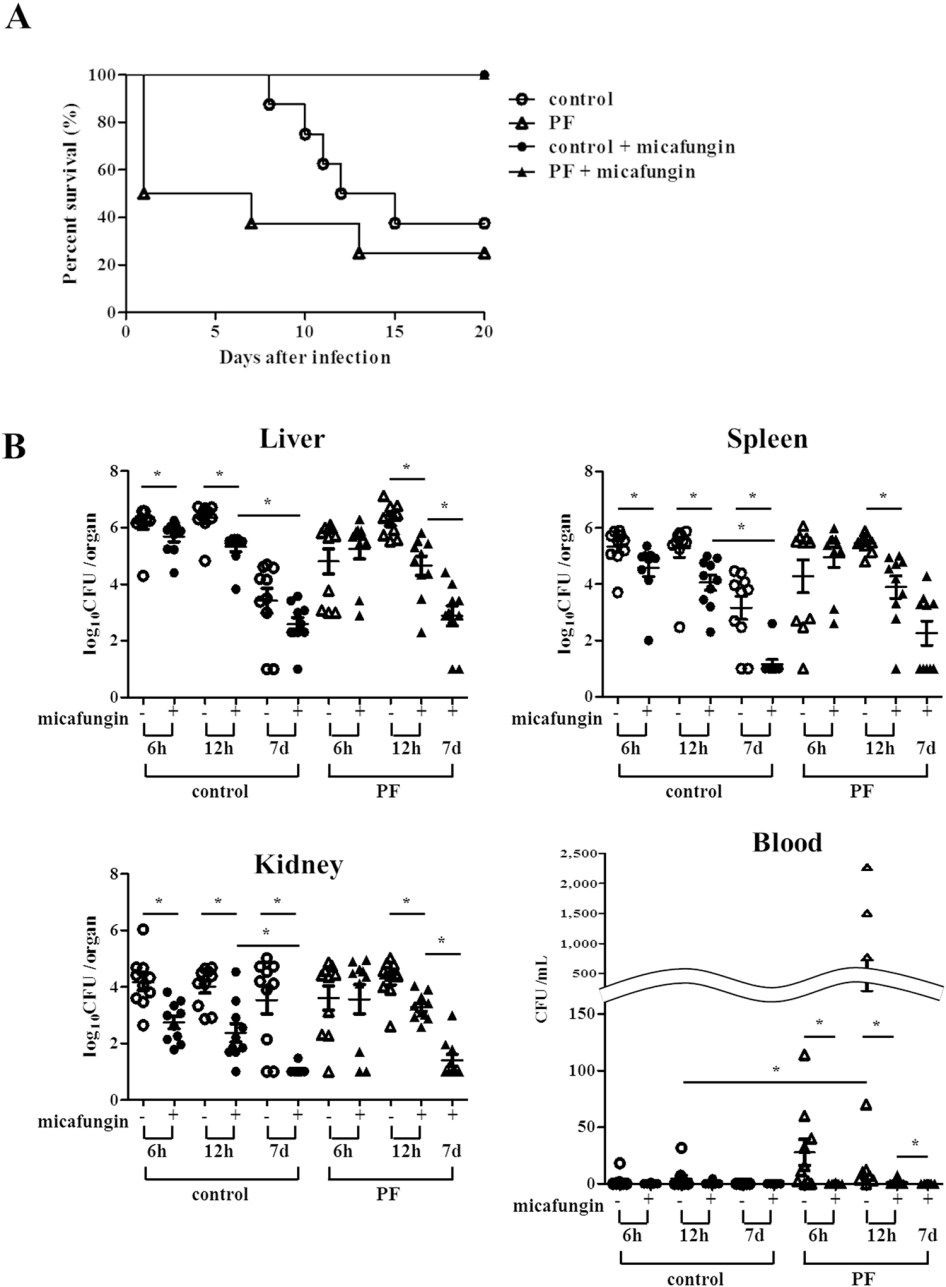
Evaluation of survival assay and the fungal burden in the liver, spleen, and kidneys. (**A**) Survival curves were plotted for the following four groups (n = 8 for each group); control without micafungin treatment (open circles); PF without micafungin treatment (open triangles); control with micafungin treatment (filled circles); and PF with micafungin treatment (filled triangles). Without micafungin treatment, the PF mice had significantly higher mortality than the control mice on day 7 (p = 0.013, Kaplan-Meier log-rank test), although both groups showed no significant difference on day 20 (p = 0.29). All the PF and control mice treated with micafungin survived until the end of the experiment (day 20) (vs. untreated group, p < 0.01 each). Similar results were obtained in three different experiments and representative data are shown. (**B**) Fungal burden in the liver, spleen, bilateral kidneys, and blood was evaluated 6 h, 12 h, and 7 days after the infection (n = 10 for each group). In the control mice, fungal burden in the three target organs at 6 and 12 h, and that in spleen and kidneys on day 7 was significantly reduced by micafungin treatment. In the PF mice, there was significant difference in the fungal burden between micafungin-treated and -untreated groups at 12 h, but not at 6 h. Fungal burdens of all the three organs in the control mice and two organs (liver and kidneys) in the PF mice were significantly reduced by micafungin treatment on day 7 compared with the results at 12 h. Asterisks indicate p < 0.0083 (Mann-Whitney U test adjusted with Bonferroni correction). PF, peritoneal fibrosis.

We evaluated survival curves of the control and PF mice with or without micafungin treatment (Fig. 2A). When untreated with micafungin, the PF mice showed a higher mortality rate than the control mice on day 7 (p = 0.013, Kaplan-Meier log-rank test) but there was no statistically significant difference in survival rate on day 20 between these two groups (p = 0.29, Kaplan-Meier log-rank test). In both PF and control groups, all mice treated with micafungin survived until the end of the experiment (day 20).

### Evaluation of the fungal burden in the liver, spleen, kidneys, and blood

The fungal burden in the liver, spleen, kidneys, and blood were evaluated at 6 h, 12 h, and 7 days after the infection (Fig. 2B). The fungal burden of the PF mice without micafungin treatment on day 7 was not assessed because majority of this group died as shown in Fig. 2A. At early timepoints, micafungin treatment significantly reduced fungal burden in all the organs examined in both control and PF mice at 12 h, but only in the control mice at 6 h. Blood stream infection (positive blood culture) was detected in some PF mice at 6 and 12 h but successfully treated with micafungin. Micafungin treatment also reduced fungal burden of spleen and kidneys in control mice on day 7 compared with no-treatment mice. In both control and PF groups with micafungin treatment, fungal burden was significantly decreased in all the organs on day 7 compared with the data at 12 h (except for spleen of PF group).

### Histopathological evaluation of *Candida* cells in the peritoneum and organs

**Figure 3:**
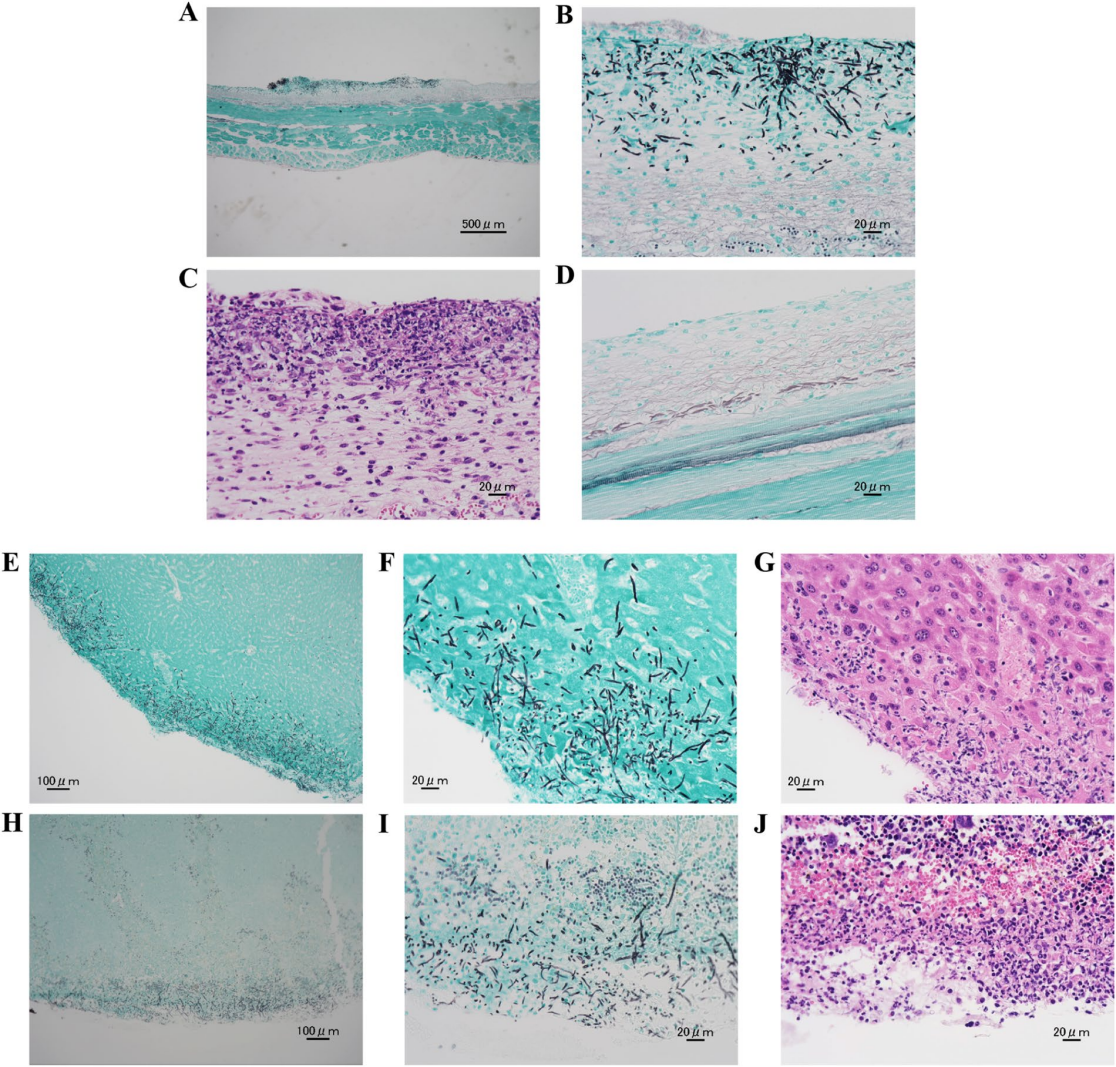
Histopathological examination of the peritoneum, liver and spleen with H & E and Grocott staining. Histopathological examination was performed 12 h after the infection. (**A**,**B**) Abscess formation with *Candida* cells in the thickened submesothelial zone of peritoneum was observed with Grocott staining only in the PF mice without micafungin treatment. (**C**) Numerous inflammatory cells were also observed with H & E staining in the same sections. (**D**) *Candida* proliferation was not detectable in the PF mice with micafungin treatment with Grocott staining. Proliferative *Candida* infiltration into the subcapsular structure inside the liver (**E**,**F**) and spleen (**H**,**I**) were observed with Grocott staining only in the PF mice without micafungin treatment. (**G**,**J**) Numerous inflammatory cells were also observed with H & E staining in the same sections. The photographs are representative of two independent examinations. Magnification, A × 40, B × 400, C × 400, D × 400, E × 100, F × 400, G × 400, H × 100, I × 400, and J × 400.

Grocott staining of the peritoneum showed abscess formation in a markedly thickened submesothelial zone with the presence of many *Candida* cells in most PF mice without micafungin treatment at 12 h after the infection (Fig. 3A,B). hematoxylin and eosin (H & E) staining confirms numerous inflammatory cells in the same sections (Fig. 3C). However, *Candida* proliferation was hardly observed in PF group with micafungin treatment (Fig. 3D). *Candida* cells were also hardly confirmed in both control groups with or without micafungin treatment probably due to the early timepoint of evaluation.

Grocott staining of the liver (Fig. 3E,F) and spleen (Fig. 3H,I) showed proliferative *Candida* infiltration into the subcapsular structure inside the organ in most PF mice without micafungin treatment at 12 h after the infection. H & E staining confirms numerous inflammatory cells in both organs (Fig. 3G,J). Adhesion of *Candida* cells to the surface of the organ was observed in some mice in both control groups (with or without micafungin treatment) and PF group (with micafungin treatment), although infiltration inside the organ was not detected in any mice in these groups. No *Candida* cell was detected in the kidneys in all the groups (photographs not shown).

### Evaluation of the cytokines in the blood

**Figure 4:**
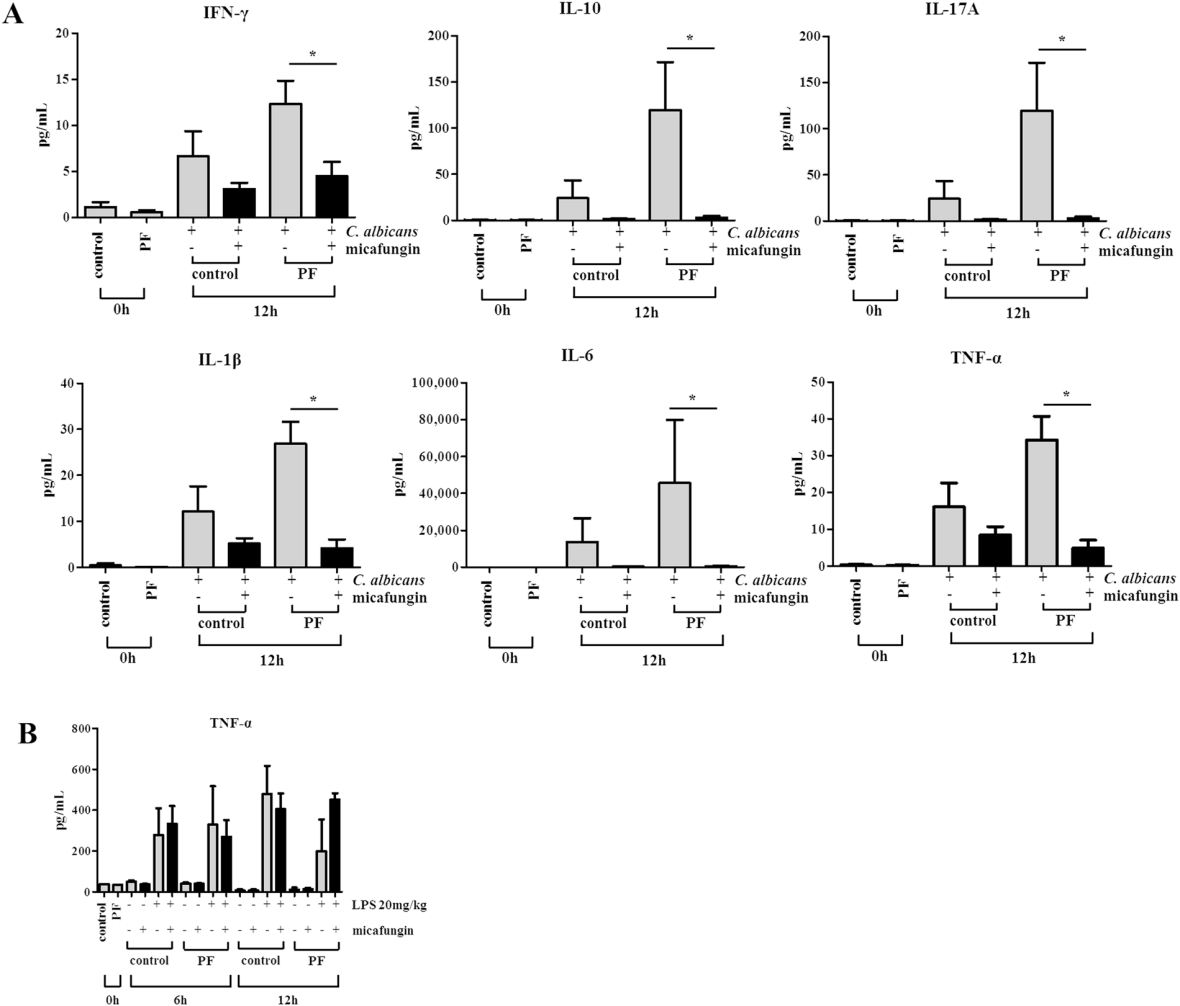
Evaluation of the cytokines in the plasma. (**A**) The plasma cytokine concentrations of IFN-γ, IL-10, IL-17A, IL-1β, IL-6, and TNF-α were quantified by enzyme-linked immunosorbent assay in the following groups: control group (control) and PF group (PF) just before infection (0 h); and infected with or without micafungin administration, in both PF and control groups at 12 h after the infection (four mice each). All the six cytokines at 12 h after the intraperitoneal inoculation of *C*. *albicans* tended to be suppressed with micafungin treatment in both PF and control groups. (**B**) The plasma TNF-α concentrations of LPS-exposed mice instead of *C*. *albicans* inoculation were quantified by enzyme-linked immunosorbent assay, and no significant difference was observed between micafungin-treated and -untreated mice in both control and PF groups. Similar results were obtained in two different experiments and representative data are shown. Asterisks indicate p < 0.05 (Mann-Whitney U test). IFN-γ, interferon-gamma; IL-10, interleukin-10; IL-17A, interleukin-17A; IL-1β, interleukin-1 beta; IL-6, interleukin-6; TNF-α, tumor necrosis factor alpha; PF, peritoneal fibrosis; and LPS, lipopolysaccharide.

We quantified the plasma cytokine concentrations to evaluate an inflammatory response in the *Candida* peritonitis mouse models. At 12 h after the infection, interferon-gamma, interleukin-10, interleukin-17A, interleukin-1 beta, interleukin-6, and tumor necrosis factor alpha (TNF-α) tended to be suppressed with micafungin treatment in both PF and control mouse models (Fig. 4A). We also used lipopolysaccharide (LPS) instead of *C*. *albicans* to evaluate if micafungin exerts an anti-inflammatory effect irrespective of the *Candida* infection. The plasma concentration of TNF-α was increased by intraperitoneal LPS injection but not suppressed with micafungin administration in both PF and control groups (Fig. 4B).

## Discussion

Long-term PD causes histopathological changes, such as PF, in the peritoneum^24^, associated with mesothelial loss, severe thickening of the submesothelial compact zone, and vascular alterations^3^. Severe or prolonged infective peritonitis also leads to PF, eventually leading to membrane^23,24^ and organ failures^28^. However, the influence of PF in the prognosis of peritonitis remains to be evaluated. In the present study, we evaluated the severity of *C*. *albicans* peritonitis in the context of PF using mouse models induced by CG exposure. Although, some differences exist between CG-induced PF and human PF with PD therapy, most of the pathological changes between them are similar. These include, expression of collagen and alpha-smooth muscle actin, macrophage infiltration, and neovascularization in the peritoneum^29^. Therefore, CG mouse models have been used to investigate the pathogenesis and therapy of PF^4,30–32^. In our study, the presence of PF enhanced early mortality in mice with *Candida* peritonitis during the first 7 days. In a histopathological evaluation, we hereby showed the proliferation of *Candida* cells, invading into the liver and spleen tissues, only in the PF group without micafungin treatment; whereas, with micafungin treatment, it was inhibited. Interestingly, no infiltration was found in all the control mice regardless of micafungin treatment. That no *Candida* cell invasion was detected histopathologically in the kidneys might have resulted from the location of these organs in the retroperitoneal space, which is separated from the intraperitoneal space. In addition, the absolute number of *Candida* cells in kidneys were approximately 100 times less than those in other two organs. The proliferation of *Candida* cells in the peritoneum was also detected only in the PF mice without micafungin treatment, histopathologically. Considering all these findings, the damage to the peritoneal membrane and a remarkable intraperitoneal *Candida* proliferation followed by candidemia, which was confirmed by the significant increase of fungal burden in the bloodstream at 12 h, are thought to have caused the early high mortality phase in the PF group.

In our study, we confirmed all mice in both PF and control groups were successfully treated with early initiation of micafungin 2 h after *C*. *albicans* inoculation based on the results of survival, fungal burden, and histopathological findings. Drug penetration into the site of infection to achieve microbe-eliminating concentrations is a key requirement for effective antimicrobial treatment. It should be noted that micafungin concentration at the site of infection may be above MICs for micafungin susceptible *Candida* species but below mutant prevention concentrations^33^. This caution is important because intra-abdominal candidiasis is a hidden reservoir for emergence of echinocandin resistance particularly in cases of *Candida glabrata* infection^34^. A limitation of the current study is that we were unable to evaluate effects of PF on drug penetration into the peritonea and peritoneal cavity.

Concerning the good efficacy of micafungin demonstrated in this study, the regulation of cytokines might also be playing an important role. During acute inflammation induced by infection in the peritoneal cavity, proinflammatory cytokines are activated during the early period, and the neutrophils recruited are subsequently replaced by monocytes^35^. While proinflammatory and anti-inflammatory cytokines are critical to the elimination of the infection, excessive production can cause tissue and organ damages^36^. It was reported that micafungin suppresses LPS-induced TNF-α production and may have immunomodulative effects^37^. In the present study, we could not confirm the suppression of plasma TNF-α concentration by micafungin administration in LPS-exposed mouse models, in both PF and control groups. Therefore, the tendency for the suppression of excessive inflammatory cytokines in *Candida* peritonitis mice by micafungin is considered to be due mainly to the secondary effect of its direct fungicidal activity against *Candida*, resulting in an improved survival rate. We considered that anti-inflammatory cytokine IL-10 was induced secondarily to inflammatory cytokine production and reduced at 12 h as a result of the suppression of inflammatory cytokines by micafungin treatment.

In conclusion, the present study demonstrated that *Candida* peritonitis itself showed a high mortality even in subjects without PF, and PF enhanced early mortality in *Candida* peritonitis. Micafungin showed a good efficacy for *Candida* peritonitis even in the context of PF. This result may also apply to patients who develop PF due to intra-abdominal inflammation caused by other reasons besides PD, such as abdominal surgery, intra-abdominal infection, and malignant diseases^25–27^. Considering the high mortality result in the PF groups during the early period, early initiation of micafungin is considered important. The *Candida* peritonitis mouse model with underlying PF developed in the present study could also be useful in future studies to evaluate the pathogenicity of other *Candida* strains and the efficacies of other antifungal agents in similar conditions.

## Materials and Methods

### Ethics

Our animal experiment protocol was pertinently reviewed and approved by the Institutional Animal Care and Use Committee of Nagasaki University (approval number 1407281164). All animal experiments were performed at the Nagasaki University Laboratory Animal Center for Biomedical Research in accordance with the Guide for the Care and Use of Laboratory Animals (National Research Council, National Academy Press, Washington DC, 2011) and the institutional regulations and guidelines for animal experimentation.

### Murine model of PF

The animals used in this study were specific-pathogen-free male ICR mice (10 weeks of age, weighing approximately 40 g; CLEA Japan, Inc., Japan). They were housed in the Biomedical Research Center, Life Science Support Center, Nagasaki University.

We induced PF by intraperitoneal administration of CG as described previously^4,30–32^. Mice received injections of 0.1% CG in 15% ethanol, or 15% ethanol alone, at a dose of 0.2 mL/mouse into the peritoneal cavity on alternate days for 3 weeks, 9 times overall.

### Evaluation of the peritoneum thickness

For the evaluation of PF, the PF and control mice were sacrificed 4 days after the last CG injection (n = 6 for each group). In order to prevent bias, peritoneal membrane was dissected in four locations (upper right, lower right, upper left, and lower left), and evaluated with Masson’s trichrome staining. Thus, four sections were analyzed for each mouse. The thickness of the submesothelial compact zone for each section was measured at 10 positions and compared between the PF and control groups, using the unpaired t test.

### Murine *Candida* peritonitis and antifungal treatment

*Candida* peritonitis was induced by intraperitoneal inoculation of the *C*. *albicans* wild-type strain SC5314. *C*. *albicans* cells grown in yeast extract-peptone-dextrose (YPD) broth overnight were washed and resuspended in sterile saline and adjusted to 5.0 × 10^7^ CFU/mL. Mice were infected intraperitoneally with 1000 µL of the *Candida* suspension 4 days after the last CG injection. Non-infected groups were intraperitoneally injected with 1000 µL of sterile saline instead of *C*. *albicans* cell suspension.

All the mice in the treatment groups were treated with 5 mg/kg of micafungin (500 µg/mL) (Astellas Pharma Inc., Tokyo, Japan) injection subcutaneously in the neck, once a day for seven days, beginning 2 h after the intraperitoneal injection of *C*. *albicans* cell suspension; based on the same administration schedule against candidiasis reported previously^38^.

The MIC of micafungin was ≤0.015 mg/L, determined by dry plate antifungal susceptibility testing of yeasts; Eiken (Eiken Chemical Co., Ltd., Tokyo, Japan). We used the subcutaneous route of administration because repeated injection into the tail vein of mice is difficult and it is reported that there is only a marginal difference in the efficacy of this drug between subcutaneous and intravenous administrations^38^. The dosage of 5 mg/kg corresponds to 125 mg/day in humans^39^ consistent with serum concentration-time curve data from 0 to 24 h (AUC_0-24_) between humans and mice^40,41^. The mice in the non-treatment groups were administered with sterile saline alone during the same period.

### Evaluation of survival, fungal organ and bloodstream burdens and histopathological examination in mice

The survival of the mice was recorded daily after the intraperitoneal injection of *C*. *albicans* until day 20 (n = 8 for each group). Statistical analysis was performed using the Kaplan-Meier method.

To assess the viable cell count of *Candida* in the organs; the liver, spleen, and bilateral kidneys were excised and placed in sterile 0.9% saline at 4 °C immediately after sacrifice, at 6 h, 12 h, and 7 days after the intraperitoneal injection of *C*. *albicans* (n = 10 for each group). The homogenate was then serially diluted 1:10, and aliquots were plated on YPD agar. Blood samples were also collected by cardiac puncture and plated on YPD agar without dilution. Viable fungal colony counts were determined after 24–48 h incubation at 30 °C. The lower limit of detection was 10 CFU/organ. The results were expressed as log_10_ CFU/organ for the three organs and CFU/mL for blood, and analyzed with the Mann-Whitney U test followed by Bonferroni correction.

For the histopathological evaluation, the peritoneum, liver, spleen, and kidney, obtained 12 h after the intraperitoneal injection of *C*. *albicans* were stained with H & E and Grocott.

### Evaluation of the cytokines in the murine blood

The plasma cytokine concentrations of interferon-gamma, interleukin-10, interleukin-17A, interleukin-1 beta, interleukin-6, and TNF-α were quantified using a Bio-Plex Pro^TM^ Mouse Cytokine Th17 Panel A 6-Plex (#m6000007ny, Bio-Rad Laboratories, Hercules, CA) for the following groups. These were, the control and PF groups just before infection; and infected with or without micafungin administration, in both PF and control groups at 12 h after the infection (four mice each).

The plasma TNF-α concentrations in mice exposed to LPS were also quantified as follows: 20 mg/kg of LPS were administered intraperitoneally, after 2 h, 5 mg/kg of micafungin (500 µg/mL) was injected subcutaneously to the treatment group. Sterile saline was subcutaneously injected to the non-treatment groups. Plasma TNF-α concentrations, obtained 6 and 12 h after LPS administration were measured using Mouse TNF alpha Uncoated ELISA (88-7324, Thermo Fisher Scientific Inc., Waltham, MA). Each of the groups consisted of four mice.

### Statistical analysis

The unpaired t test was used to assess differences of the PF thickness. The differences of fungal burden in the target organs and the cytokines in the plasma were evaluated by using the Mann-Whitney U test. Multiple comparisons were adjusted with the Bonferroni method. For all statistical analyses, a value of p < 0.05 was considered significant. All statistical analyses were performed using Prism 5.0 (GraphPad Software, Inc., La Jolla, CA).
